# Endophytic strategies decoded by genome and transcriptome analysis of *Fusarium nematophilum* strain NQ8GII4

**DOI:** 10.3389/fmicb.2024.1487022

**Published:** 2025-01-15

**Authors:** Siyuan Yan, Qingchen Zhang, Shuxin Jia, Miaomiao Guo, Qiangqiang Zhang, Peiwen Gu

**Affiliations:** ^1^College of Forestry and Prataculture, Ningxia University, Yinchuan, China; ^2^Department of Pharmacotherapy and Translational Research, University of Florida, Gainesville, FL, United States; ^3^School of Agriculture, Ningxia University, Yinchuan, China

**Keywords:** *Fusarium nematophilum* strain NQ8GII4, fungal-plant mutualistic associations, comparative genomics, transcriptome, endophytic strategy

## Abstract

**Introduction:**

*Fusarium nematophilum* strain NQ8GII4 is an endophytic fungus with significant potential for improving growth and disease resistance of alfalfa. However, the molecular mechanisms underlying the symbiotic relationship between NQ8GII4 and alfalfa roots remain poorly understood.

**Methods:**

In this study, we conducted (1) a comparative genomic analysis of selected saprophytic, pathogenic, and endophytic fungi, including molecular phylogeny analysis, whole-genome alignment, and divergence date estimation positioning, and (2) transcriptomic profiling of alfalfa roots infected with NQ8GII4.

**Results:**

Our findings reveal that NQ8GII4 is genetically closely related to *F. solani*, suggesting it diverged from *Fusarium* phytopathogens. During the early stages of symbiosis establishment, genes encoding glycosyltransferases (GTs), fungal cell wall-degrading enzymes (FCWDEs), and steroid-14α-demethylase (CYP51) were significantly downregulated, potentially suppressing hyphal growth of the fungus. Once symbiosis was established, NQ8GII4 secreted effectors that activated plant immunity, which in turn could slow growth of the fungus. Moreover, genes involved in secondary metabolite biosynthesis, such as type I polyketide synthases (T1PKS) and non-ribosomal peptide synthetases (NRPSs), were significantly downregulated. Homologs of autophagy-related genes, including *ATG1, ATG2, ATG11*, and others, were also downregulated, suggesting that reduced phytotoxin production and autophagy inhibition is a consequence of NQ8GII4’s symbiosis.

**Discussion:**

This study investigated the comprehensive molecular and genetic mechanisms governing the interaction between NQ8GII4 and alfalfa roots. Beyond the NQ8GII4-alfalfa system, these findings also provide a valuable molecular framework for understanding the mechanism of interactions between endophytic fungi and their host plants.

## 1 Introduction

Fungi can be classified as endophytes, pathogens, and saprophytes (Zhou et al., 2018). Endophytes are microorganisms that can inhabit healthy plant tissue temporarily or permanently without causing disease (Tian et al., 2022). In symbiotic relationships, endophytes can promote host plant growth, produce secondary metabolites, and enhance plant stress tolerance by influencing plant physiology and gene expression. In return plants provide endophytes with nutrients and shelter (Manganiello et al., 2018; Rozpadek et al., 2015). The potential of endophytes in agricultural applications has been widely recognized. Endophytes and pathogens can have close phylogenetic relationships (Xu et al., 2014). The growth rate of endophyte within the plant is crucial for maintaining mutualistic relationships. Disruption of processes required to establish and/or maintain the mutualistic relationship can transform an endophyte into a pathogen (Jiang, 2015). Eaton et al. (2015) identified a core set of 182 genes associated with mutualism in *Epichloë* spp. The set of genes includes upregulated genes encoding degradative enzymes, transporters and primary metabolic proteins as well as downregulated genes encoding small secreted proteins and secondary metabolic enzymes. Inactivation of one or combinations of these genes has potential to disrupt the mutualistic relationship. Rahnama et al. (2018) observed global regulatory factor VelA negatively regulates the colonization of *E. festucae* in *Lolium perenne* (ryegrass). *ΔvelA* could invade the vascular bundles leading to a high level of ryegrass seedlings dying. Green et al. (2019) demonstrated *E. festucae* without PlsA (homolog of Pls1) had a proliferative pattern of growth within the leaves of ryegrass. Ryegrass seedlings infected with the *ΔplsA* had a reduced survival rate.

The endophytic fungus *Fusarium nematophilum* strain NQ8GII4 (hereafter NQ8GII4) was successfully isolated from the roots of healthy wolfberry, and could form mutualistic relationships with various plants, such as *Medicago sativa* L. (alfalfa), and *Triticum aestivum* L. (wheat) (Hu et al., 2020; Siyuan et al., 2020; Zhang et al., 2022). Previous studies have demonstrated that NQ8GII4 exhibits growth-promoting activities, including siderophore-production, phosphate solubilization, and the secretion of gibberellin, cellulase, and protease (Li J. et al., 2023). The NQ8GII4 strain colonizes the roots of wolfberry, inducing defensive responses in the plant and enhancing its resistance to root rot (Yan et al., 2024). When NQ8GII4-GFP colonized the roots of alfalfa, it initiated transcriptional reprogramming in the plant. The NQ8GII4 enhanced the activities of nitrate reductase (NR) and glutamine synthetase (GS) in alfalfa by modulating photosynthesis and the JA signaling pathway. This led to the accumulation of soluble sugars and proteins in the plants (Jia, 2024). However, the molecular mechanisms underlying the endophytic relationship between the NQ8GII4 and alfalfa are not yet fully understood.

To further understand the symbiotic interaction between NQ8GII4 and alfalfa, we sequenced the genome of NQ8GII4 and conducted a comparative study with the genomes of four endophytes [*E. festucae*, *S. indica*, *Pestalotiopsis fici*, *F. oxysporum* strain Fo47 (hereafter Fo47)], five pathogens [*Verticillium dahliae*, *Pyricularia oryzae*, *F. solani*, *F. oxysporum* strain Fol4287 (hereafter Fol4287), *Colletotrichum higginsianum*], and two saprophytes (*Aspergillus nidulans*, *Neurospora crassa*). The comparative genomic analyses focused on key gene categories among fungi with different lifestyles to address the question, Do endophytes have a specific genomic signature? Additionally, we compared the transcriptomes of NQ8GII4 in free-living mycelia and in symbiotic alfalfa tissues to identify symbiosis-related genes. These research findings will elucidate the endophytic strategies employed by NQ8GII4, which will provide a theoretical basis for understanding mechanisms underlying beneficial plant-fungus interactions.

## 2 Materials and methods

### 2.1 Fungal and plant material

The endophytic fungus NQ8GII4 was isolated by the Laboratory of Plant Pathology, School of Agriculture, Ningxia University, and deposited at China General Microbiological Culture Collection Center (CGMCC No. 19271). The strain was maintained on potato dextrose agar (PDA) medium and lyophilized filter paper for short and long term storage. For subsequent assays, this strain was growing on a PDA medium for 5 d at 25°C. The *Agrobacterium tumefaciens* strain GV3101 (hereafter GV3101) was used for agroinfiltration of plants, cultured on lysogeny broth medium at 28°C.

Gannon No. 3 is a variety of alfalfa, widely cultivated in Gansu, Ningxia, Qinghai, and Xinjiang Province in western China (Zhang, 2018), and was purchased from Ningxia Shanggu Agricultural and Animal Husbandry Development Co. (Ningxia, China). *Nicotiana benthamiana* seedlings were grown in a glasshouse at 14 h 22°C: 10 h 20°C, day: night, 72% relative humidity.

### 2.2 NQ8GII4 genome sequencing and assembly

Three 5 mm NQ8GII4 mycelial disks were placed in Czapek medium and incubated on a rotary shaker at 175 rpm at 25°C for 4 days. The genomic DNA of NQ8GII4 was extracted from the mycelia using the Omage Fungal DNA Kit. Purified genomic DNA was quantified by TBS-380 fluorometer (Turner Biosystems Inc., Sunnyvale, CA). Short insert library (400 bp) was constructed and sequenced on an Illumina HiSeq X Ten Sequencing System (Shanghai Majorbio Bio-Pharm Technology Co., Ltd.), generating 150 bp paired-end reads. In order to make the subsequent assembly more accurate, low quality bases and sequencing adapter sequences were trimmed and filtered from the raw illumina reads using Trimmomatic v. 0.36 (Bolger et al., 2014). SOAPdenovo2 (Luo et al., 2012) was tested to assemble the NQ8GII4 genome. The gene set integrity was then evaluated with BUSCO v5.4.5 (Simão et al., 2015) and CEGMA v2.5 (Parra et al., 2007). The assembled genome sequence was deposited into the GenBank database under accession No. JAVIXY000000000.1.

### 2.3 Gene prediction and annotation

The annotation of the genome of NQ8GII4 was performed with MAKER2 v2.31 (Holt and Yandell, 2011), Snap, Augustus v2.5.5,^1^ and Evidence Modeler.^2^ The protein sequences of the predicted genes were compared with NCBI Non-Redundant (NR), Kyoto Encyclopedia of Genes and Genomes (KEGG), Swiss-Prot protein, Pfam, Cluster of Orthologous Genes (COG), and Gene Ontology (GO) databases by BLASTp (BLAST+2.9.0 with *E*-value cut-off <1e−5). The repeats were identified by RepeatMasker v4.1.4.^3^ barrnap V0.9^4^ and tRNAscan-SE v2.0 (Lowe and Eddy, 1997) were used to predict the rRNA and tRNA contained in the genome.

### 2.4 Orthologous genes and evolution analysis

OrthoVenn 3 (Sun et al., 2023) was used to analyze the orthologs pairs of 5 endophytes (NQ8GII4, *E. festucae*, *S. indica*, *P. fici*, Fo47), 5 pathogens (*V. dahliae*, *P. oryzae*, *F. solani*, Fol4287, *C. higginsianum*), and 2 saprophytes (*A. nidulans*, *N. crassa*) (Supplementary Table 1). The tools were executed with the *E*-value cutoff of 1e−5 for all-to-all protein similarity comparisons, and an inflation value of 1.5 for the generation of orthologous cluster using the Markov Cluster Algorithm. For evolutionary divergence data estimation, clustering, protein family selection and phylogenetic analysis were performed with OrthoVenn 3.

### 2.5 NQ8GII4 inoculation manipulation

Three 5 mm NQ8GII4 mycelial disks were placed in Czapek medium and incubated on a rotary shaker at 175 rpm at 25°C for 4 days, the fermentation broth of containing 10^7^ CFU/mL of NQ8GII4 growth was obtained. To avoid the effect of microorganisms other than NQ8GII4 on gene expression in alfalfa. Seeds of healthy Gannon No.3 were cleaned in sterile water, rinsed with 75% ethanol for 30 s, and sterilized with 10% NaClO for 1 h, and washed four times with sterile water. Excess water was removed using sterile filter paper, and then the seeds were added to 70 mL of Hoagland’s nutrient solution in a 500 mL bottle. The seeds were germinated inside the bottles under the following conditions: 22°C with a light intensity of 4000 lx and a photoperiod of 16 h light and 8 h dark. After 25 days of incubation, the roots of the resulting seedlings were inoculated with 15 mL of fermentation broth containing 10^7^ CFU/mL of NQ8GII4 growth. All operations were carried out under sterile conditions, and the plants were grown in an incubator with 20 replicates.

### 2.6 cDNA library construction and RNA-seq

According to the infestation process of NQ8GII4 in the alfalfa root (Supplementary Figure 1), alfalfa seedlings were harvested at 0.5, 1, 6, and 14 days post inoculation (dpi), three biological replicates were collected at each treatment, and fifteen whole plants were pooled as a single replicate. The obtained plants were rapidly frozen with liquid nitrogen and maintained at −80°C until RNA was extracted. A1, A2, and A3 are alfalfa samples of NQ8GII4 inoculation at 12 hpi, B1, B2, and B3 are alfalfa samples of NQ8GII4 inoculation at 1 dpi, C1, C2, and C3 are alfalfa samples of NQ8GII4 inoculation at 6 dpi, D1, D2, and D3 are alfalfa samples of NQ8GII4 inoculation at 14 dpi, E1, E2, and E3 are mycelia of NQ8GII4.

Samples of A1 ∼ E3 total RNA were extracted using E.Z.N.A.^®^ Total RNA Isolation Kit (Omega, USA) according to the manufacturer’s recommendations. After integrity, quality, and purity checking, the purification of messenger RNA (mRNA), construction of complementary DNA (cDNA) libraries, cDNA end repair, adapter ligation, and cDNA amplification were performed by Majorbio Technologies Co., Ltd. (Shanghai, China). The final quantified libraries were sequenced on an Illumina Hiseq 2000 platform. RNA-Seq data generated in this study were mapped to the NQ8GII4 genome using HISAT2 (Kim et al., 2015), and transcriptome-based gene structures were obtained by String Tie (Pertea et al., 2015). RNA-Seq data with accession PRJNA1125526 were deposited in the NCBI databases.^5^

### 2.7 Identification of differential expression genes (DEGs)

FPKM (fragments per kilobase of exon model per million mapped reads) (Trapnell et al., 2010) was used to standardize the expression levels. With the aid of the DESeq program (Love et al., 2014), differential expression analysis of A/B/C/D-vs-E was carried out. The cut-off value of *P*-value < 0.01 and at least 2 fold change (FC) was set as threshold for differential expression.

### 2.8 Protein-encoding gene family classification

The identification and annotation of carbohydrate active enzymes (CAZymes) were performed on the dbCAN3 meta server (*E*-value < 1e−15, coverage > 0.35) (Zheng et al., 2023). Transporters, secreted peptidases, and G-protein-coupled receptors (GPCR) were annotated by a BLAST search of the Transporter Classification Database (TCDB) v2020091 (Saier et al., 2021), MEROPS (Rawlings et al., 2014), and GPCRdb (Pándy-Szekeres et al., 2023) using a BLASTp E-value cut-off of 1E−5. GPCR characterized by a seven transmembrane domain structure, and GPCR-like proteins were evaluated to verify the presence of seven transmembrane helices using TMHMM 2.0 (Krogh et al., 2001). GPCR-like proteins functional annotation was performed by blasting with the Pathogen Host Interaction (PHI) database (Urban et al., 2022). Cytochrome P450s were analyzed on the online tool at Majorbio Cloud Platform.^6^ SignalP V4.1 (Petersen et al., 2011), TMHMM 2.0, Big-PI Fungal Predictor (Eisenhaber et al., 2004), ProtComp V9.0 (Emanuelsson et al., 2000), and EffectorP v3.0 (Sperschneider and Dodds, 2022) were used to distinguish effector proteins in the NQ8GII4 genome. Gene clusters related to secondary metabolite biosynthesis were identified using the antibiotic and secondary metabolite analysis shell (antiSMASH V7.0) (Blin et al., 2021) online tool. The parameter setting was maintained as the default parameter values.

### 2.9 Quantitative real-time PCR (qRT-pCR) analysis

Quantitative, real-time RT-PCR (qRT-PCR) assays were conducted with the same total RNA samples that were used for RNA-seq studies. We chose 10 DEGs at random for qRT-PCR investigation to confirm the accuracy of the RNA-Seq data. *TUB* was selected as an internal standard to calculate the relative expression levels. This analysis was conducted using the qTOWER 2.2 system (Jena, Germany) with TB Green Premix *Ex Taq* II FAST qPCR mix (Takara, Japan). qRT-PCR was carried out with 12.5 μL of 2 × TB Green Premix *Ex Taq* II FAST qPCR mix, 1.0 μL of sense and antisense primers (2.5 μM), 1.0 μL of cDNA in a final volume of 25 μL. The cycling program was set as follows: 94°C for 10 min, followed by 40 cycles of 94°C for 5 s, and 60°C for 10 s. Each plate was repeated thrice in independent runs for all reference and selected genes. Data were analyzed using qPCRsoft 4.1 quantitative PCR software. All primers are listed in the Supplementary Table 2. Primers were synthesized by Sangon Biotech Co., Ltd. (Shanghai, China). The 2^–ΔΔCT^ method was used for relative quantification of gene expression and transformed into log_2_FC.

### 2.10 Functional analysis of putative effector proteins

Coding regions of 25 genes encoding putative effectors with signal peptides (SPs) were amplified from a NQ8GII4 complementary DNA (cDNA) library with gene-specific primers (Supplementary Table 2) using Hieff Canace^®^ Plus High-Fidelity DNA Polymerase (Yeasen, China). The amplicons were subsequently ligated with potato virus X (PVX) vectors (ClaI and SalI for PVX vector). All constructs were validated by sequencing in Sanger (Sanger Biotech, Shanghai, China).

The constructs were transformed into GV3101 using hot shock method. After selection with selective antibiotics, individual colonies validated by PCR were cultured in lysogeny broth medium at 28°C in a shaking incubator at 220 rpm for 48 h. The bacteria were then pelleted by centrifugation and suspended in MES buffer (10 mM magnesium chloride (MgCl_2_), 10 mM MES, 200 μM acetosyringone, pH 5.7) in the dark for 3 h at room temperature (RT) before infiltration. For infiltration, suspended GV3101 cells were adjusted to a final OD600 of 0.6. GV3101 cell suspension was infiltrated into plant leaves using a syringe without a needle. To determine cell-death-inducing activity of the proteins, PVX constructs were agroinfiltrated into plants. Symptom development was monitored visually 5 d post agroinfiltration (dpa). GV3101 transformed with BAX1, and INF1 were used as a positive control. GV3101 transformed with PVX was used as negative control. In addition, the bacterial suspension were infiltrated into *N. benthamiana* 12 h before infiltrating GV3101 bearing the INF1 or BAX1-expressing construct.

### 2.11 Statistical analysis

A Maximum Likelihood tree was generated with amino acid sequences of A, KS, and effector with the program MEGA v.11 using the Jones Taylor Thornton model (JTT), 100 replicates for bootstrap analysis. A volcano plot was drawn by Hiplot.^7^ Heat map, stacked bar chart, circular bar plot and phylogenomic tree were drawn by Chiplot^8^ (Xie et al., 2023; Li J. et al., 2023; Ji et al., 2022). The venn diagram was drawn by TBtools (Chen et al., 2023).

## 3 Results

### 3.1 Genome sequencing and general features

**FIGURE 1:**
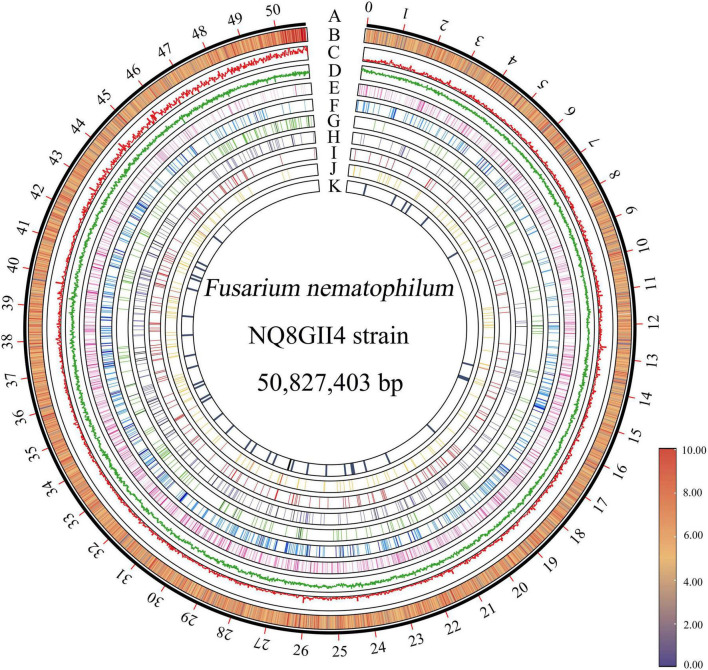
Circos plot of genome features of *F. nematophilum* strain NQ8GII4. (A) Genome size of *F. nematophilum* strain NQ8GII4. (B) Gene density (20 Kb moving average window). Distribution of GC content (C), transposable elements (D), transporter genes (E), CAZyme genes (F), CYPs (G), effector genes (H), GPCRs (I), secreted peptidases (J) and secondary metabolite clusters (K).

The genome of NQ8GII4 was assembled from the data generated by the Illumina sequencing platform. After quality control of raw data, 4,997.1 Mb data were generated. The assembled NQ8GII4 genome consisted of 2,064 scaffolds with N50 length of 144.83 Kb and a combined size of 50,827,403 bp (48.47 Mb) (Supplementary Table 3). The average GC ratio was 53.93%. A total of 15,149 protein-coding genes were predicted with a length of 28,820,034 bp. The average gene length was 1902.44 bp, and the gene density was 307 genes per 1 Mb of the genome (Figure 1). These predicted genes account for 56.70% of the genome. About 14,279 (94.25%), 10,276 (67.83%), 9,605 (63.40%), 9,428 (62.23%), 5,665 (37.40%), and 4,404 (29.07%) of the predicted genes presented homologies with known functions in the NR, Pfam, Swiss-Model, GO, COG, and KEGG databases, respectively. In addition, 233 tRNA and 50 rRNA were predicted in the assembly genome.

The repeat elements was 5.36% (2,727,425 bp) in the NQ8GII4 genome, including 4.72% (2,400,760 bp) as interspersed repeats and 0.64% (326,665 bp) as tandem repeats. The most abundant repetitive elements was the DNA transposons (1.42%, 720,961 bp), followed by long terminal repeat (LTR) (0.27%, 138,531 bp), non-LTR retrotransposons LINEs (long interspersed nuclear elements) (0.39%, 199,653 bp), rolling circle (RC) (0.72%, 367,651 bp). Among the tandem repeats, a total 7,526 (321,471 bp) microsatellite were identified, accounting for 0.63% of the genome. In addition, 41 (0.01%, 5,194 bp) satellite were identified. 4,414 disrupted genes were identified in NQ8GII4 genome, suggesting that the repeat elements have had an important influence on the dynamics of the evolution of the NQ8GII4 genome (Supplementary Table 4).

### 3.2 Comparative genomic analysis

**FIGURE 2:**
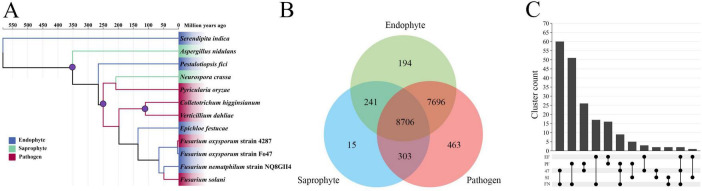
Phylogenomic relationships and ortholog groups in *F. nematophilum* strain NQ8GII4 and 11 other fungal species. **(A)** Maximum likelihood (ML) phylogenetic tree was constructed showing the evolutionary relationships and diveragence dates of 5 endophytic (blue), 5 pathogenic (red), and 2 saprophytic (green) species. **(B)** Orthologs among pathogens, endophytes, and saprophytes. **(C)** Orthologs among five endophytes. The values explain the counts of the orthologs groups. EF, *E. festucae*; PF, *P. fici*; SI, *S. indica*; FN, *F. nematophilum* strain NQ8GII4; 47, *F. oxysporum* strain Fo47.

The phylogeny was calibrated using three calibration points. *A. nidulans* and *N. crassa* diverged approximately 300 ∼ 400 MYA, *P. oryzae* and *C. higginsianum* diverged approximately 207 ∼ 339 MYA, *V. dahliae* and *C. higginsianum* diverged approximately 100 ∼ 150 MYA (Hacquard et al., 2016; O’Connell et al., 2012). NQ8GII4 and *F. oxysporum* diverged approximately 60 ∼ 70 MYA, NQ8GII4 and *F. solani* diverged relatively recently (45 ∼50 MYA), suggested that NQ8GII4 is closely related to *F. solani* (Geiser et al., 2021). It also suggested that each lifestyle has evolved independently of the others (Figure 2A).

An orthovenn 3.0 analysis identified 17,618 ortholog groups (127,204 genes) that were clustered between NQ8GII4 and the other 11 fungal genomes [comprising phytopathogens (*V. dahliae*, *P. oryzae*, *F. solani*, Fol4287, and *C. higginsianum*), endophytes (*E. festucae*, *S. indica*, *P. fici*, and Fo47), and saprophytes (*A. nidulans*, and *N. crassa*)]. On average, each ortholog group contained approximately 7.22 genes. No clusters were identified that were shared by all symbiotic fungi, but were absent from the genomes of pathogens and saprophytes (Figures 2B, C).

### 3.3 Gene family descriptions

#### 3.3.1 Carbohydrate active enzymes (CAZymes)

CAZYmes are enzymes responsible for breaking down complex carbohydrates and polysaccharides into smaller products, promoting the growth and pathogenicity of microorganisms. According to the catalytic activity, CAZYmes were classified into glycoside hydrolases (GHs), polysaccharide lyases (PLs), carbohydrate esterases (CEs), glycosyltransferases (GTs), carbohydrate-binding modules (CBMs), and auxiliary activities (AAs) (Wenxiu et al., 2021). According to the substrate, CAZYmes were divided into five classes: cellulase (CAZYmes families including GH5, GH6, GH7, GH12, GH45, AA9/GH61, and CBM1), hemicellulase (CAZYmes families including GH10, GH11, GH26, GH27, GH31, GH35, GH36, GH43, GH51, GH53, GH54, GH62, GH93, CE1, CE2, CE3, CE5, CE12, CE15, and CE16), pectase (CAZYmes families including GH28, GH78, GH88, GH95, GH105, PL1 ∼ 4, PL 9 ∼ 11, and CE 8), ligninase (CAZYmes families including AA 1 ∼ 8), fungal cell wall-degrading enzymes (FCWDEs, CAZYmes families including GH16, GH17, GH18, GH20, GH55, GH64, GH71, GH72, GH75, GH76, GH81, GH85, GH92, CE4, CBM12, CBM14, CBM18, CBM19, CBM24, CBM43, and CBM52) (Lyu et al., 2015; Veneault-Fourrey et al., 2014; Zhao et al., 2014).

**FIGURE 3:**
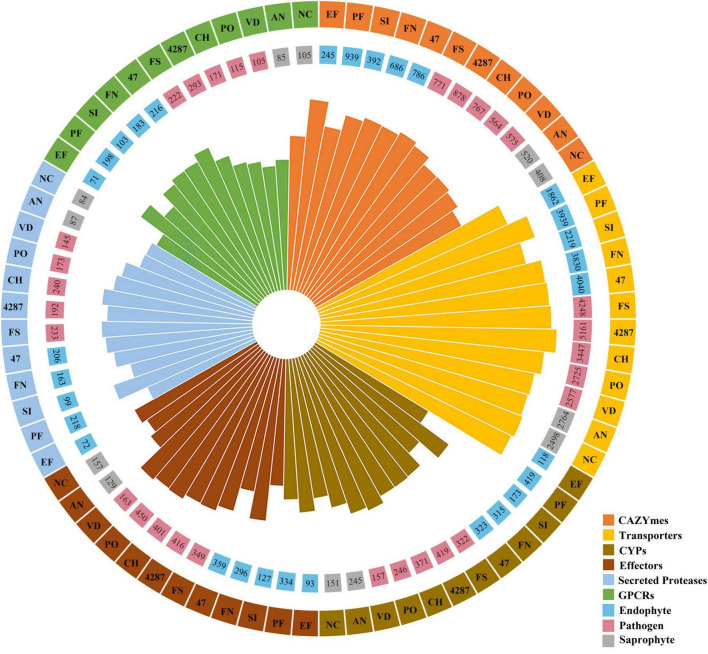
Families of genes encoded in 12 fungal genomes examined in this study. In the center, color represented different gene families. The height of wedge indicates the gene number of different gene families in 12 fungal examined. The gene number enriched in each gene families are shown in the inner circle. In the inner circle, color indicate the fungus with different lifestyles. In the outer circle, color represent different gene families. The strains are shown in the outer circle, EF, *E. festucae*; PF, *P. fici*; SI, *S. indica*; FN, *F. nematophilum* strain NQ8GII4; 47, *F. oxysporum* strain Fo47; FS, *F. solan*; 4287, *F. oxysporum* strain Fol4287; CH, *C. higginsianum*; PO, *P. oryzae*; VD, *V. dahliae*; AN, *A. nidulans*; NC, *N. crassa*.

**FIGURE 4:**
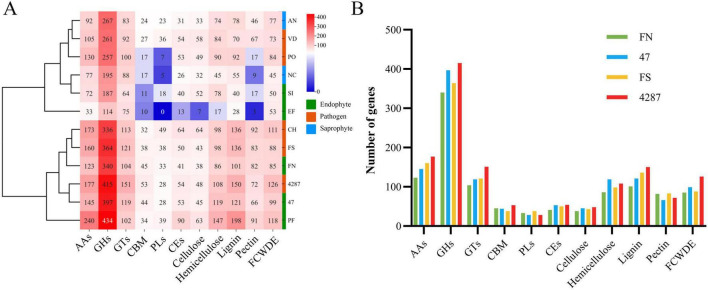
CAZYmes of NQ8GII4 **(A)** Comparison of composition of CAZYmes in 12 fungal genomes. **(B)** Comparison of CAZYmes composition in 4 *Fusarium* species. FN, *F. nematophilum* strain NQ8GII4; 47, *F. oxysporum* strain Fo47; FS, *F. solan*i; 4287, *F. oxysporum* strain Fol4287.

The NQ8GII4 genome included 686 CAZymes modules. This number was within the range of numbers of CAZymes modules in the five pathogens (564 – 878, average 717) and other endophytes (245 – 939, average 610). The NQ8GII4 genome encoded more CAZymes modules than the 2 saprophytes examined (408 – 520, average 464) (Figure 3). Based on the profile of function and substrates of CAZymes, the NQ8GII4 was similar to Fo47, Fol4287, *F. solani*, *P. fici*, and *C. higginsianum*, while endophytes *S. indica* and *E. festucae* were similar to *A. nidulans*, *N. crassa*, *V. dahliae*, and *P. oryzae* (Figure 4A). In a comparison of all five endophytes, NQ8GII4 was most similar to Fo47, strains had fewer genes encoding lignin degrading enzymes compared to *F. solani* and Fol4287 (Figure 4B).

#### 3.3.2 Membrane transporters

**FIGURE 5:**
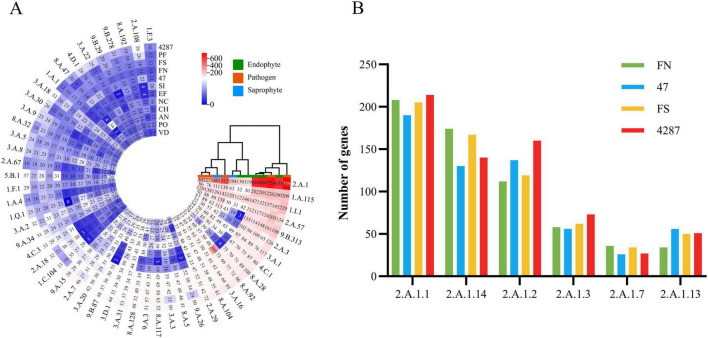
Membrane transporters of NQ8GII4. **(A)** Comparison of composition of transporters in 12 fungal genomes. **(B)** Comparison of MFS composition in 4 *Fusarium* species. FN, *F. nematophilum* strain NQ8GII4; 47, *F. oxysporum* strain Fo47; FS, *F. solan*i; 4287, *F. oxysporum* strain Fol4287.

Membrane transporters constitute a diverse group of proteins that form the contained intricate networks of channels, carriers, pumps, group translocators, and electron flow carriers that determine the molecular compositions and energy status of cells (Saier et al., 2016). The NQ8GII4 genome included 3,830 genes encoding membrane transporters. This number was within the range of numbers of membrane transporters genes in the five pathogens (2,583 – 5,161, average 3,632) and other endophytes (1,867 – 4,048, average 3,015). The NQ8GII4 genome encoded more transporters than the two saprophytes examined (2,503 – 2,772, average 2,631) (Figure 4). Based on the profile of the top 50 most abundant transporter families, the NQ8GII4 was similar to Fo47, Fol4287, *F. solani*, and *P. fici*, while endophytes *S. indica* and *E. festucae* were similar to *A. nidulans*, *N. crassa*, *C. higginsianum*, *V. dahliae*, and *P. oryzae*. In comparisons of transporter genes in the other *Fusarium* strains examined, NQ8GII4 was most similar to Fo47 and least similar to *F. solani*. The results suggest that transporters exert influence on plant-microorganism interactions to some degree (Figure 5A). Major facilitator superfamilies (MFS) couple the transportation of substrates to proton motive force generated across the cell membrane. MFS transporters target a wide spectrum of substrates including lipids, ions, amino acids and peptides, carbohydrates, and nucleosides (Chen et al., 2021). The most enriched membrane transporter family in NQ8GII4 is the MFS. A total of 694 predicted MFS transporters of 25 classes were identified in the genome of NQ8GII4. Drug: H+ Antiporter (DHA) transporters are predicted to play important roles in multidrug resistance and cell physiology (Ma et al., 2022). When compared with *F. solani*, NQ8GII4 exhibited a reduction in the abundance of DHA1 (2.A.1.2) and DHA2 (2.A.1.3) (Figure 5B).

#### 3.3.3 Cytochrome P450s (CYPs)

**FIGURE 6:**
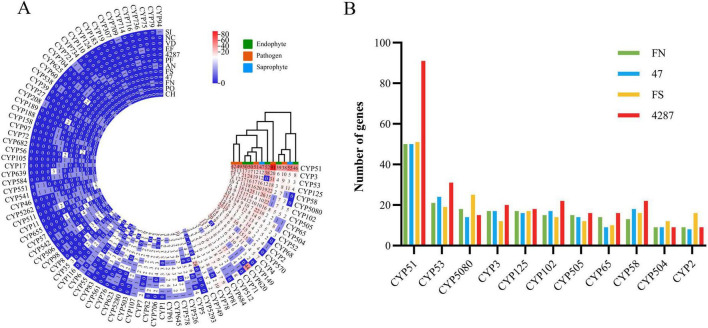
CYPs of NQ8GII4. **(A)** Comparison of composition of CYPs in 12 fungal genomes. **(B)** Comparison of composition of CYPs in 4 *Fusarium* species. FN, *F. nematophilum* strain NQ8GII4; 47, *F. oxysporum* strain Fo47; FS, *F. solan*i; 4287, *F. oxysporum* strain Fol4287.

CYPs are involved in many essential cellular processes and play diverse roles in detoxification, and biosynthesis of secondary metabolites (Xiao et al., 2012). The NQ8GII4 genome included 183 genes encoding CYPs. This number was within the range of numbers of CYPs genes in the five pathogens (157 – 419, average 303) and other endophytes (173 – 419, average 358). The NQ8GII4 genome encoded more CYPs than the two saprophytes examined (151 – 245, average 198) (Figure 4). Based on the profile of the top 50 most abundant CYPs families, NQ8GII4 was similar to Fo47, *P. fici*, Fol4287, *F. solani*, *C. higginsianum*, and *P. oryzae*, while endophytes *S. indica* and *E. festucae* were similar to *A. nidulans*, *N. crassa*, and *V. dahliae*. Among the other *Fusarium* strains examined, NQ8GII4 was most similar to Fo47 (Figures 6A, B).

#### 3.3.4 G protein-coupled receptors (GPCRs)

**FIGURE 7:**
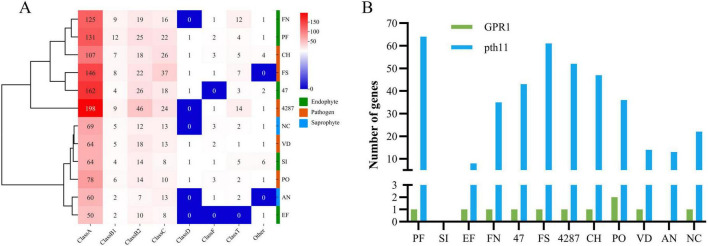
GPCRs of NQ8GII4. **(A)** Comparison of composition of GPCRs in 12 fungal genomes. **(B)** Comparison of pth11-like GPCRs and GPR1 in 12 fungal species. EF, *E. festucae*; PF, *P. fici*; SI, *S. indica*; FN, *F. nematophilum* strain NQ8GII4; 47, *F. oxysporum* strain Fo47; FS, *F. solan*i; 4287, *F. oxysporum* strain Fol4287; CH, *C. higginsianum*; PO, *P. oryzae*; VD, *V. dahliae*; AN, *A. nidulans*; NC, *N. crassa*.

Signals from a diverse range of extracellular signaling molecules can be relayed by GPCRs to intracellular downstream signaling pathways to ultimately elicit cellular responses (Bi et al., 2019). The NQ8GII4 genome included 183 genes encoding GPCRs. This number was within the range of numbers of GPCR genes in the five pathogens (105 – 293, average 181) and other endophytes (71 – 216, average 181). The NQ8GII4 genome encoded more GPCRs than 2 saprophytes examined (85 – 105, average 95) (Figure 4). GPCRs were classified into eight classes: A, B1, B2, C, D1, F, T, and others. The GPCR profile of NQ8GII4 was similar to Fo47, *P. fici*, Fol4287, *F. solani*, and *C. higginsianum*, while endophytes *S. indica* and *E. festucae* were similar to *N. crassa*, *A. nidulans*, *V. dahliae*, and *P. oryzae* (Figure 7A). Pth11 homologues that are required for appressorium differentiation in response to host surface stimuli and pathogenesis (Zhang et al., 2014). Except for *S. indica*, other fungal possessed pth11 like GPCRs. A total of 35 pth11-like GPCRs were found in the NQ8GII4 genome, whereas 43, 61, and 52 were found in the Fo47, *F. solani* and Fol4287 genome. GPR1, a sucrose/glucose sensing receptor, might impact the adaptation of fungi to their niche for host infestation (Zhang et al., 2014). Each of the four *Fusarium* genomes examined had only one GPR1 ortholog (Figure 7B).

#### 3.3.5 Secreted peptidase

**FIGURE 8:**
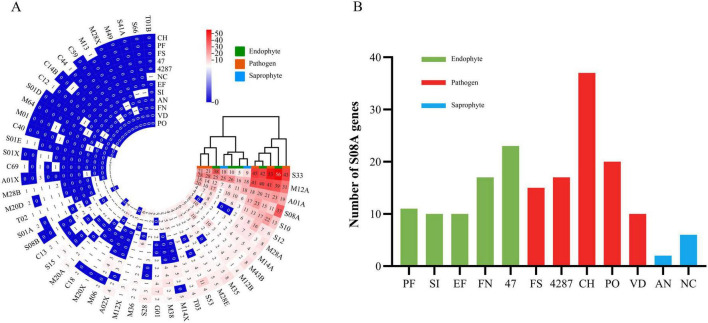
Secreted peptidases of NQ8GII4. **(A)** Comparison of composition of secreted peptidases in 12 fungal genomes. **(B)** Comparison of S08A in 12 fungal species. EF, *E. festucae*; PF, *P. fici*; SI, *S. indica*; FN, *F. nematophilum* strain NQ8GII4; 47, *F. oxysporum* strain Fo47; FS, *F. solan*i; 4287, *F. oxysporum* strain Fol4287; CH, *C. higginsianum*; PO, *P. oryzae*; VD, *V. dahliae*; AN, *A. nidulans*; NC, *N. crassa*.

Peptidases, also known as proteinases, are responsible for protein degradation. In fungi, secreted peptidases could function in both nutrition and degradation of host defense proteins, and could thus interfere with plant innate immunity (Ehsan et al., 2020; Lopez et al., 2018). The NQ8GII4 genome included 163 genes encoding secreted peptidases. This number was within the range of numbers of secreted peptidase genes in the five pathogens (145 – 240, average 197) and other endophytes (72 – 218, average 149). The NQ8GII4 genome encoded more secreted peptidases than the two saprophytes that were examined (84 – 87, average 86) (Figure 4). These peptidases were classified into six superfamilies: serine peptidase, metallo peptidase, cysteine peptidase, aspartic peptidase, threonine peptidase, and glutamic peptidase superfamilies. The secreted peptidase profile of NQ8GII4 was similar to *S. indica*, *E. festucae, N. crassa*, *A. nidulans*, *V. dahliae*, and *P. oryzae*, while endophytes Fo47 and *P. fici* were similar to Fol4287, *F. solani*, and *C. higginsianum* (Figure 8A). Subtilisins (S08A), a family of serine proteases associated with virulence, penetration and colonization of hosts (Knapp et al., 2018). The NQ8GII4 genome encoded 17 S08A, similar to the 5 pathogens examined (ranging from 10 to 37, average 19.8) and the 4 endophytes examined (ranging from 10 to 23, average 13.5), encodes more S08A than the 2 saprophytes examined (ranging from 2 to 6, average 4) (Figure 8B).

#### 3.3.6 Effector

**FIGURE 9:**
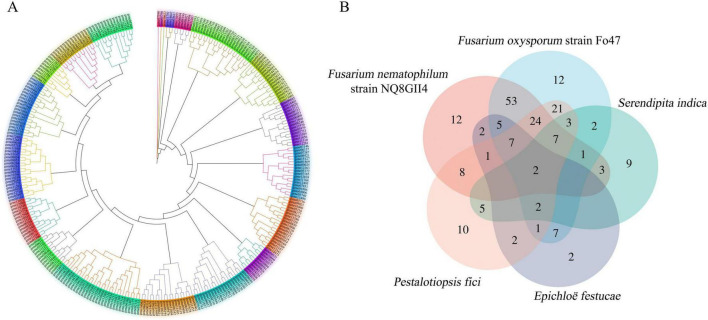
Effectors of NQ8GII4. **(A)** The phylogeny of the 296 putative effectors in NQ8GII4. **(B)** Venn diagram showing orthologs between the five endophytes effectors.

Microorganisms secrete an arsenal of effectors to modulate host innate immunity and enable infection (Johnson et al., 2013). The NQ8GII4 genome included 296 genes encoding putative effectors (Figure 9A). This number was within the range of numbers of putative effector genes in the five pathogens (163 – 463, average 197) and other endophytes (93 – 359, average 228). The NQ8GII4 genome encoded more effectors than the 2 saprophytes examined (129 – 157, average 143) (Figure 4). Two ortholog groups were shared by NQ8GII4 and other endophytes (Figure 9B), which were lower than pathogens (14) (Supplementary Figure 2A), and saprophytes (9) (Supplementary Figure 2B). In addition, 69 ortholog groups were shared by NQ8GII4 and the three other *Fusarium* strains examined (Supplementary Figure 2C), showing a high level of conservation in the adaptation to host plants among the four *Fusarium* strains.

#### 3.3.7 Secondary metabolite gene clusters

Some secondary metabolites produced by plant-associated fungi can affect pathogenicity and/or host range (Wang et al., 2017). Forty-one putative secondary metabolite biosynthetic gene clusters were detected in the NQ8GII4 genome, including 9 non-ribosomal peptide synthetases (NRPS), 10 NRPS-like, 9 type I polyketide synthases (T1PKS), 4 terpene, 2 fungal post-translationally modified peptide like (fungal-RiPP-like), 2 type III polyketide synthases (T3PKS), phosphonate, indole, betalactone, NI siderophore, and NRPS-T1PKS gene cluster has one. Compared to Fo47, the secondary metabolites gene cluster profile of NQ8GII4 is similar to *F. solani*, but there are also some differences. The NQ8GII4 genome encoded more T3PKS, NI siderophore, and betalactone gene cluster, while encoding less T1PKS, NRPS, indole, terpene, and isocyanide gene cluster (Supplementary Figure 3).

**FIGURE 10:**
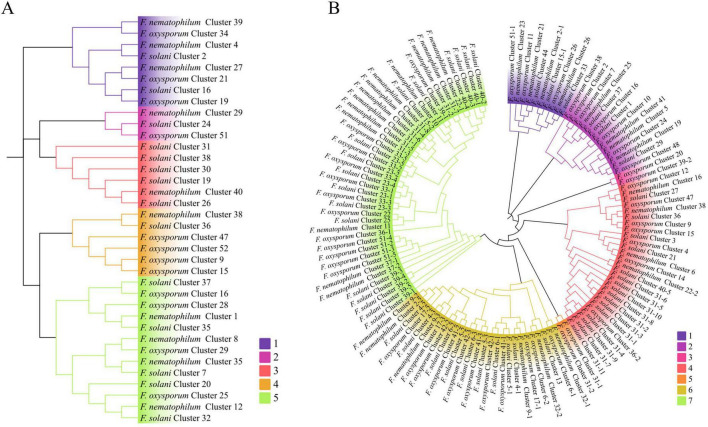
Phylogenetic analysis of PKS **(A)** and NRPS **(B)** from NQ8GII4, Fo47 and *F. solani* based on the KS and A domain sequence. The colors represented different clade of PKS and NRPS.

NRPSs and PKSs were the most frequently occurring secondary metabolite gene clusters in the NQ8GII4, *F. solani*, and the Fo47. The phylogenetic relationships among NRPSs, PKSs were analyzed based on the A or KS domain sequences (Figure 10). The KS domain phylogenetic analysis indicated that the PKSs from these three strains could be grouped in 5 clades, and the NQ8GII4 encoded fewer PKSs in clade 3. The A domain phylogenetic analysis indicated that the NRPSs from the three compared strains could be grouped in 7 clades, and the NQ8GII4 encoded fewer NRPSs in clade 4, 6, and 7. The reduction of these genes may result in the decreased pathogenicity of NQ8GII4.

### 3.4 Transcriptome analyses of NQ8GII4 during symbiosis with alfalfa

To understand transcriptional reprogramming during the mutualistic symbiosis of NQ8GII4 and alfalfa, gene profiles of NQ8GII4 were studied during infection at 0.5, 1, 6, and 14 dpi. In total, 2,091 genes were identified as DEGs. Among these genes, 100 (7 upregulated and 93 downregulated), 143 (75 upregulated and 68 downregulated), 597 (56 upregulated and 541 downregulated), and 1,714 (661 upregulated and 1,053 downregulated) were identified in 0.5, 1, 6, and 14 dpi. Groups of 8, 57, 282, and 1,413 were identified as unique to 0.5, 1, 6, and 14 dpi. A total of 52 DEGs were commonly expressed at 0.5, 1, 6, and 14 dpi (Supplementary Figure 4A).

To validate the reliability of the RNA-Seq results, we randomly selected 10 DEGs for qRT-PCR analysis. The trends in expression levels of the selected genes were similar to those found in the RNA-Seq, indicating the reliability of the DEGs analyzed by RNA-Seq (Supplementary Figure 4B).

**FIGURE 11:**
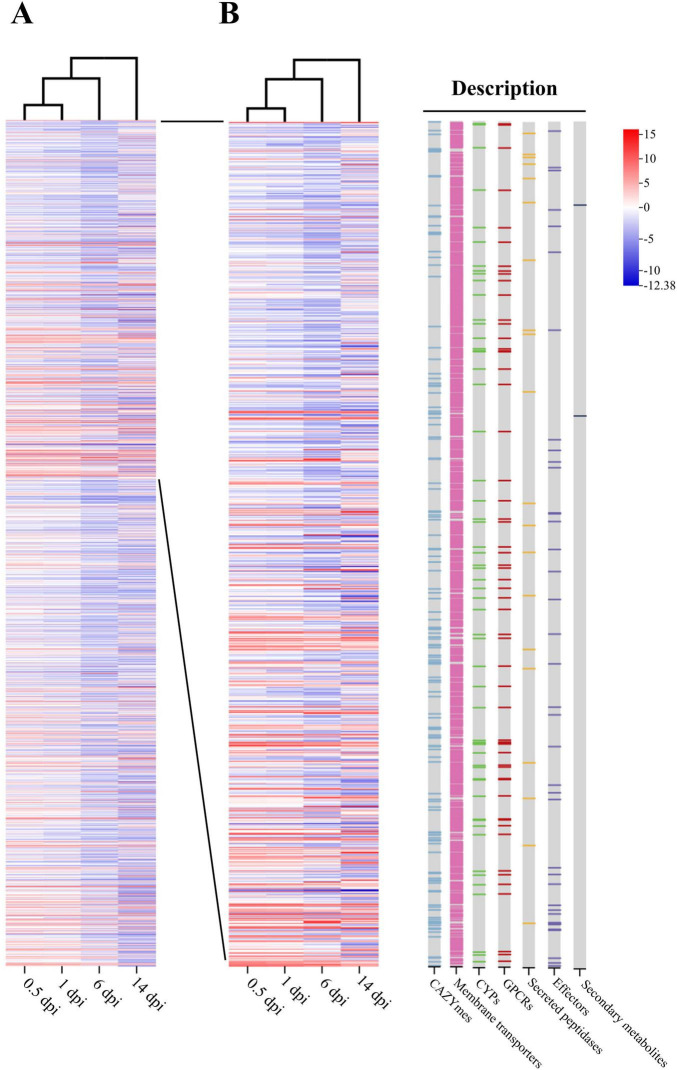
Transcript profiling of DEGs during NQ8GII4 symbiosis with alfalfa at 0.5, 1, 6, and 14 dpi. **(A)** Transcript profiling of 2,091 DEGs. **(B)** Transcript profiling of DEGs related to CAZYmes, membrane transporters, CYPs, GPCRs, secreted peptidases, effectors, and core biosynthetic gene of secondary metabolite.

The gene profile of NQ8GII4 at 0.5 dpi was similar to 1 dpi. Downregulated DEGs were mainly enriched in GTs, FCWDEs, S08A, steroid-14α-demethylase (CYP51), and the sugar porter family. One DHA1 and one DHA2 were upregulated at 1 dpi. Gene09380, annotated as GH12 protein, a putative cellulase (Wang et al., 2018), was upregulated at 1 dpi, suggesting that NQ8GII4 begins infecting by degrading the alfalfa cell wall. At 6 dpi, upregulated DEGs were enriched in plant cell wall-degrading enzymes (PCWDEs), such as enzymes degrading cellulose, hemicellulose, and pectin. At 14 dpi, upregulated DEGs were enriched in the group of effectors, and CYPs BM-3 (CYP102). Two different PTH11-like GPCRs were significantly upregulated at 6 and 14 dpi, respectively. These findings suggest that NQ8GII4 respond to different environment signals at 6 and 14 dpi. The core biosynthetic gene of secondary metabolite cluster 9 (NRPS) were downregulated at 0.5, 1, 6, and 14 dpi, and the core biosynthetic gene of secondary metabolite cluster 4 (T1PKS) was significantly downregulated at 14 dpi. 3, 12, and 31 putative effector genes were upregulated at 1, 6, and 14 dpi, respectively. Of these putative effector genes, 1, 5, and 11 genes upregulated at 1, 6 and 14 dpi, respectively, were also annotated as CAZyme-encoding genes. Futhermore, autophygy related genes, such as gene08729 (homolog of *ATG1*), gene00631 (homolog of *ATG2*), gene07294 (homolog of *ATG11*), and others, were downregulated at 6 and 14 dpi (Figure 11).

### 3.5 Functional studies on putative effectors

**FIGURE 12:**
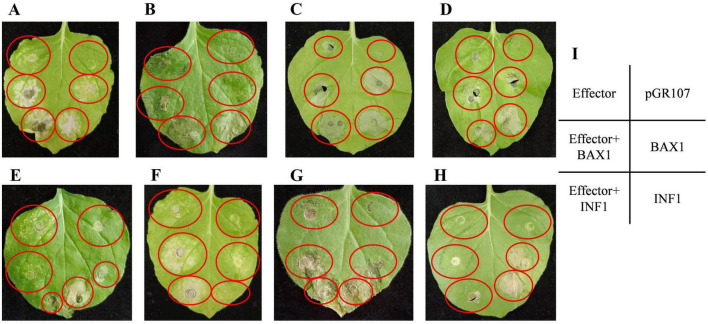
*Agrobacterium*-mediated expression of NQ8GII4 effector in *N. benthamiana*. **(A–G)** FnEG1, FnEG3, FnEG7, FnEG8, FnEG168, FnEG214, and FnEG218 could triggered cell death in *N. benthamiana*. **(H)** FnEG100 could suppress INF or BAX-triggered plant cell death. **(I)** Schematic diagram of *Agrobacterium*-mediated expression of NQ8GII4 effector in *N. benthamiana*.

We carried out transient expression of 25 full-length effectors in *N. benthamiana*. The positive control using vector containing the BAX or INF gene, and PVX vector was used as the negative control. It was shown that, FnEG1 (Gene00104), FnEG3 (gene00314), FnEG7 (gene00635), FnEG8 (gene00639), FnEG168 (gene10147), FnEG214 (gene11857), and FnEG218 (gene11959) could trigger cell death in *N. benthamiana*. By contrast, PVX vector did not induce plant cell death, suggesting the cell death was significantly activated by these effectors. BAX and INF strongly induced plant cell death at 5 d post inoculation (dpi), whereas FnEG100 (gene07554) could fully suppress INF or BAX-triggered plant cell death (Figure 12). FnEG1, FnEG7, FnEG8, FnEG100, FnEG168, FnEG214, and FnEG218 contained AA9, CFEM, Cerato-platanin, SUN, AltA, Pectata lyase, and GH16 domain, respectively (Supplementary Figure 5). In RNA-seq analysis of NQ8GII4-infected alfalfa roots, *FnEG168* was upregulated at 1 and 14 dpi, while *FnEG1*, *FnEG8*, and *FnEG214* were upregulated at 14 dpi.

## 4 Discussion

NQ8GII4 is an endophytic fungus isolated from the roots of healthy wolfberry, and can form mutualistic relationships with multiple plant species. Endophytes exhibited the characteristic of growth promoting and disease resistance enhancing (Hu et al., 2020; Siyuan et al., 2020; Zhang et al., 2022; Jia, 2024). Laser confocal microscopy revealed that NQ8GII4, like other general endophytes such as endophytes of *F. oxysporum* (Redkar et al., 2022a) and *Serendipita indica* (Redkar et al., 2022b), primarily colonizes the root surface and cortex (Supplementary Figure 1). This colonization pattern is distinct from *Fusarium* phytopathogens, which extensively colonize the root cortex and vasculature (Yang et al., 2024). Thus, we sequenced the genome of NQ8GII4 and conducted a comparative analysis with the genomes of four endophytes, five pathogens, and two saprophytes. To elucidate the endophytic molecular mechanisms of the NQ8GII4, we monitored gene expression during NQ8GII4-alfalfa interaction using RNA-seq.

### 4.1 NQ8GII4 diverged from *Fusarium* phytopathogens

Molecular phylogeny, whole-genome alignment, and divergence date estimates indicate that 5 endophytes examined evolved independently from each other. We then compared gene families — such as CAZymes, transporters, CYPs, GPCRs, secreted peptidases, effectors, and secondary metabolite gene clusters—among the 12 fungi studied, we found different endophytes possessed different structures of gene family. The gene family profiles of *E. festucae* and *S. indica* were similar to those of saprophytes. *Epicholoë* could act as saprophytes to enhance nutrient cycling in grassland ecosystems (Song et al., 2021). *S. indica*, displaying both biotrophic and saprotrophic characteristics, is particularly useful for studying the evolution from saprotrophism to biotrophism (Lahrmann et al., 2015). Endophytes like NQ8GII4, Fo47, and *P. fici* share similarities with pathogens. *P. fici* was first identified as a pathogen of *Ficus carica*. Mycoviruses and the expansion of pectinase encoding genes might have participated in the transition of lifestyles (Wang et al., 2015; Wang et al., 2023). *F. oxysporum* causes vascular wilt diseases in over 100 different crops (Constantin et al., 2020). The nonpathogenic strain Fo47 is the most studied biocontrol strain of *F. oxysporum* (Aimé et al., 2013). Redkar et al. (2021) demonstrated that pathogenic and nonpathogenic *F. oxysporum* strains share a set of conserved pathogenicity factors, underscoring that the nonpathogenic *F. oxysporum* strains are closely related to pathogenic strains. Notably, NQ8GII4 and *F. solani* are closely related taxa that diverged 45 to 50 MYA. We speculated NQ8GII4 evolved from a *Fusarium* phytopathogen ancestor.

### 4.2 The NQ8GII4-alfalfa mutualistic relationship was determined by a finely tuned equilibrium between fungal virulence and plant defense

GTs are enzymes that build complex carbohydrates from activated sugar donors and are involved in essential activities of fungal cells, such as cell wall synthesis, the glycogen cycle, and the trehalose cycle (O’Connell et al., 2012). The fungal cell wall is primarily composed of chitin, glucans, mannans, and glycoproteins. Fungal cell wall-degrading enzymes (FCWDEs), which are responsible for the breakdown or modification of the fungal cell wall, are crucial for fungal development (Lyu et al., 2015). CYP51, known as sterol-14α-demethylase, is a critical enzyme in ergosterol biosynthesis, influencing cell membrane fluidity and hyphal morphology (Tsybruk et al., 2023). The classes of GTs, CYP51, and FCWDEs enriched abundant downregulated DEGs at 0.5, 1, and 6 dpi. Given the roles of GTs, CYP51 and FCWDEs in fungal biology, this down regulation could play a role in maintaining the balance of the mutualistic relationship by preventing overgrowth of the fungus.

Microorganisms have the ability to produce a vast array of bioactive secondary metabolites, most of which are derived from a polyketide and/or non-ribosomal peptide parent compound(s). Formation of a polyketide parent compound is catalyzed by a PKS, while formation of a non-ribosomal parent compound is catalyzed by an NRPS (Hansen et al., 2015). Luo et al. (2020) discovered the deletion of the *V. dahliae* NRPS gene *VdNPS* reduced virulence of the fungus on cotton and tobacco. Lu et al. (2022) demonstrated the *Curvularia lunata* PKS gene *Clpks18* was required for production of the toxin methyl 5-(hydroxymethyl) furan 2-carboxylate as well as wild-type levels of virulence of the fungus on maize. In the current study, the core biosynthetic gene of secondary metabolite cluster 9 (NRPS) were downregulated at 0.5, 1, 6, and 14 dpi, and the core biosynthetic gene of secondary metabolite cluster 4 (T1PKS) was significantly downregulated at 14 dpi. We speculate that this may be related to the synthesis of some pathogenic toxins. However, additional experiments are required to determine whether suppression of toxin production contributes to the endophytic lifestyle of NQ8GII4.

Plants have developed two primary defense systems to counteract microbial attacks. The first, known as MAMP-triggered immunity (MTI), involves the recognition of microbe-associated molecular patterns (MAMPs) by pattern recognition receptors (PRRs) (Rebaque et al., 2021). Successful pathogens can disrupt MTI by delivering effectors, leading to effector-triggered susceptibility (ETS) (Ngou et al., 2022). In response, plants deploy resistance proteins (R) to detect these effectors, initiating effector-triggered immunity (ETI). This dynamic interaction creates a balance between plants and microorganisms (Dodds and Rathjen, 2010).

The putative NQ8GII4 effectors FnEG1, FnEG8, FnEG168, and FnEG214 triggered cell death in *N. benthamiana* in a *Agrobacterium*-mediated transient expression assay. The corresponding genes were upregulated at 14 dpi in infected alfalfa roots. FnEG1, FnEG8, FnEG168, and FnEG214 contained AA9, Cerato-platanin, AltA, and Pectata lyase domain, respectively. The function of these proteins in triggering plant immunity has been widely reported. MoHrip1, a member of the AltA family, is recognized as a MAMP by the plant immune system. When tobacco plants were treated with MoHrip1, they exhibited necrosis, increased reactive oxygen species, and upregulated defense-related genes (PR1a, PR5, HSR203J, and HIN1), along with enhanced resistance to *Botrytis cinerea* and *Pseudomonas syringae* pv. *Tabaci* (Zhang et al., 2017). The recombinant proteins of Epl-1 (Cerato-platanin) from *Trichoderma asperellum* ACCC30536 could alter signal transduction pathways of SA, JA, and IAA in *Populus davidiana* × *P. alba* var. *pyramidalis* (PdPap), promoting seedling growth and pathogen resistance against *Alternaria alternata* (Yu et al., 2018). Purified VdPEL1 (pectate lyase) triggered defense responses and conferred resistance to *B. cinerea* and *V. dahliae* in tobacco and cotton plants (Yang et al., 2018). Zarattini et al. (2021) treated Arabidopsis with *Thermothielavioides terrestris* purified AA9 protein. The phenomenon, such as an increased level of ethylene, jasmonic and salicylic acid hormones, along with deposition of callose in the cell wall, was observed. Therefore, we speculate that the establishment of the mutualistic relationship between NQ8GII4 and alfalfa involves production, by the fungus, of effectors that trigger plant immunity, which in turn slows growth of the fungus.

### 4.3 Autophygy might related to the endophytic characteristics of NQ8GII4

Autophagy is a programmed cell degradation mechanism that is ubiquitous in eukaryotic cells. Organisms can remove and degrade excess biological macromolecules and self-damaged cells that are produced in biological processes through autophagy and use the degradation products to provide energy. In fungi, autophagy plays an important role in regulating growth, development, and pathogenicity (Jiao et al., 2021). Wang et al. (2022) demonstrated that *FvAtg4* and *FvAtg8* contribute to *F. verticillioides* pathogenicity to maize by regulating the autophagic pathway to control lipid turnover, fumonisin biosynthesis, and pigmentation during its infectious cycle. Jiao et al. (2022) concluded that the *SsAtg1* gene of *Sclerotia sclerotiorum* is involved in response to nutritional stresses that govern mycelial growth and is essential for sclerotia formation, contributing to virulence and the development of compound appressoria. In our study, a large number of autophagy-related genes, such as *ATG1*, *ATG2*, *ATG11*, and others. were downregulated at 6 and 14 dpi. This finding suggests that the autophagy pathway was inhibited during the process of NQ8GII4 symbiosis. Therefore, we hypothesized the endophytic characteristics of NQ8GII4 might be related to the inhibition of autophagy pathway.

## 5 Conclusion

In summary, we sequenced the genome of *F. nematophilum* strain NQ8GII4, and performed a comparative study with the sequenced genomes of 4 endophytic, 5 pathogenic, and 2 saprophytic fungi. Analysis of ortholog groups, divergence date, and gene category indicate that endophytes evolved independently from each other, and NQ8GII4 might possess a specific endophytic mechanism. To understand endophytic transcriptional reprogramming, gene profiles of NQ8GII4 were studied during symbiosis with alfalfa at different stages. In the early stages of the symbiotic relationship establishment between the NQ8GII4 and alfalfa, the growth of hyphae NQ8GII4 was inhibited. After the symbiotic relationship has been established, NQ8GII4 secreted a large amount of effectors that triggered plant immunity. This further inhibited the excessive expansion of the NQ8GII4. In addition, genes related to secondary metabolites synthesis and autophagy, were downregulated. This suggests that the establishment of endophytic growth of NQ8GII4 in alfalfa could involve suppression of phytotoxin production and inhibition of autophagy.

## Data Availability

The datasets presented in this study can be found in online repositories. The names of the repository/repositories and accession number(s) can be found in this article/Supplementary material.

## Footnotes

1
http://augustus.gobics.de/

2
https://sourceforge.net/p/evidencemodeler/mailman/message/31423107/

3
http://www.repeatmasker.org/

4
https://github.com/tseemann/barrnap/

5
https://www.ncbi.nlm.nih.gov/bioproject/1125526

6
https://cloud.majorbio.com/page/tools/

7
https://hiplot.com.cn

8
https://www.chiplot.online/#BioPlot
