# Supplementary material for: Endophytic strategies decoded by genome and transcriptome analysis of Fusarium nematophilum strain NQ8GII4
Source: Front Microbiol. 2025 Jan 15;15:1487022. doi: 10.3389/fmicb.2024.1487022 (PMC11774914; doi:10.3389/fmicb.2024.1487022)
Supplement: Supplementary file 2 [file Data_Sheet_1.pdf]

Supplementary Material

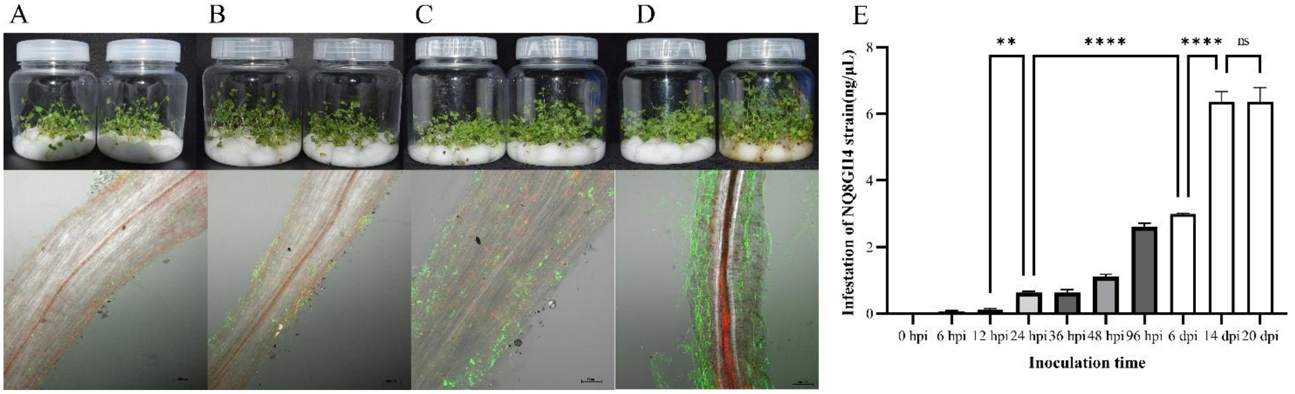

Supplementary Figure 1. Colonization of *F. nematophilum* strain NQ8GII4 in alfalfa roots (A) The alfalfa roots were co-cultivated with NQ8GII4 for 12 hours (B) The alfalfa roots were co-cultivated with NQ8GII4 for 1 day (C) The alfalfa roots were co-cultivated with NQ8GII4 for 6 days (D) The alfalfa roots were co-cultivated with NQ8GII4 for 14 days. The samples were dyed using WGS; the red and green fluorescence came from the roots of alfalfa and NQ8GII4 respectively. The left bottle was control, and the right bottle was treatment of NQ8GII4. (E) Determination of NQ8GII4 infestation in alfalfa root samples

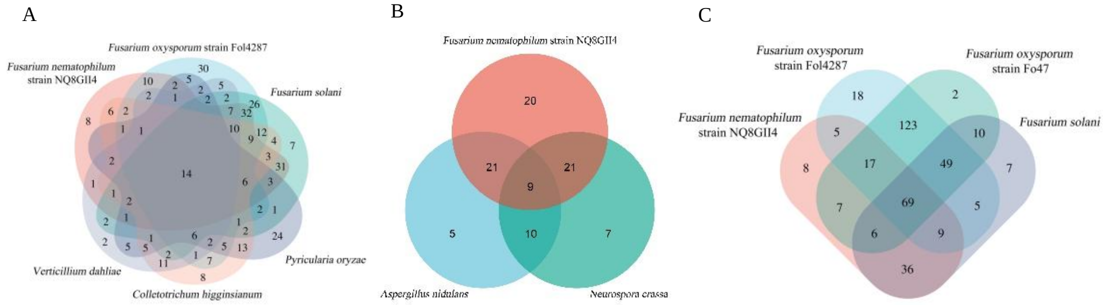

Supplementary Figure 2. Venn diagram showing orthologs among *F. nematophilum* strain NQ8GII4 and 6 pathogen fungal (A), 2 saprophyte (B), and 3 Fusarium. The value explain the counts of the ortholog groups.

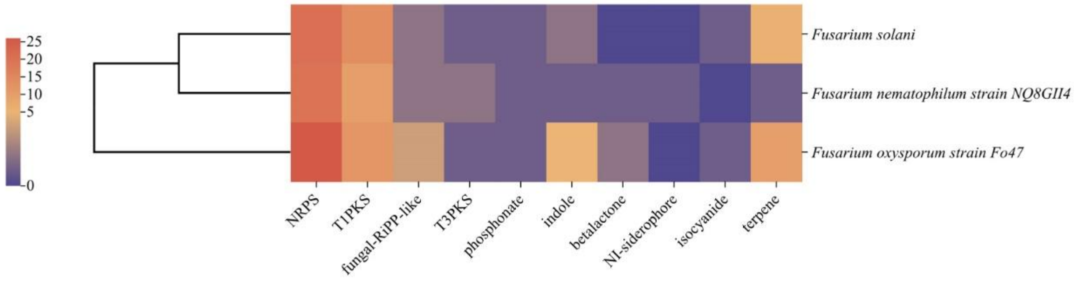

Supplementary Figure 3. Distribution of secondary metabolite gene clusters encoded in genomes of *F. nematophilum* strain NQ8GII4, *F. oxysporum* strain Fo47, and *F. solani*.

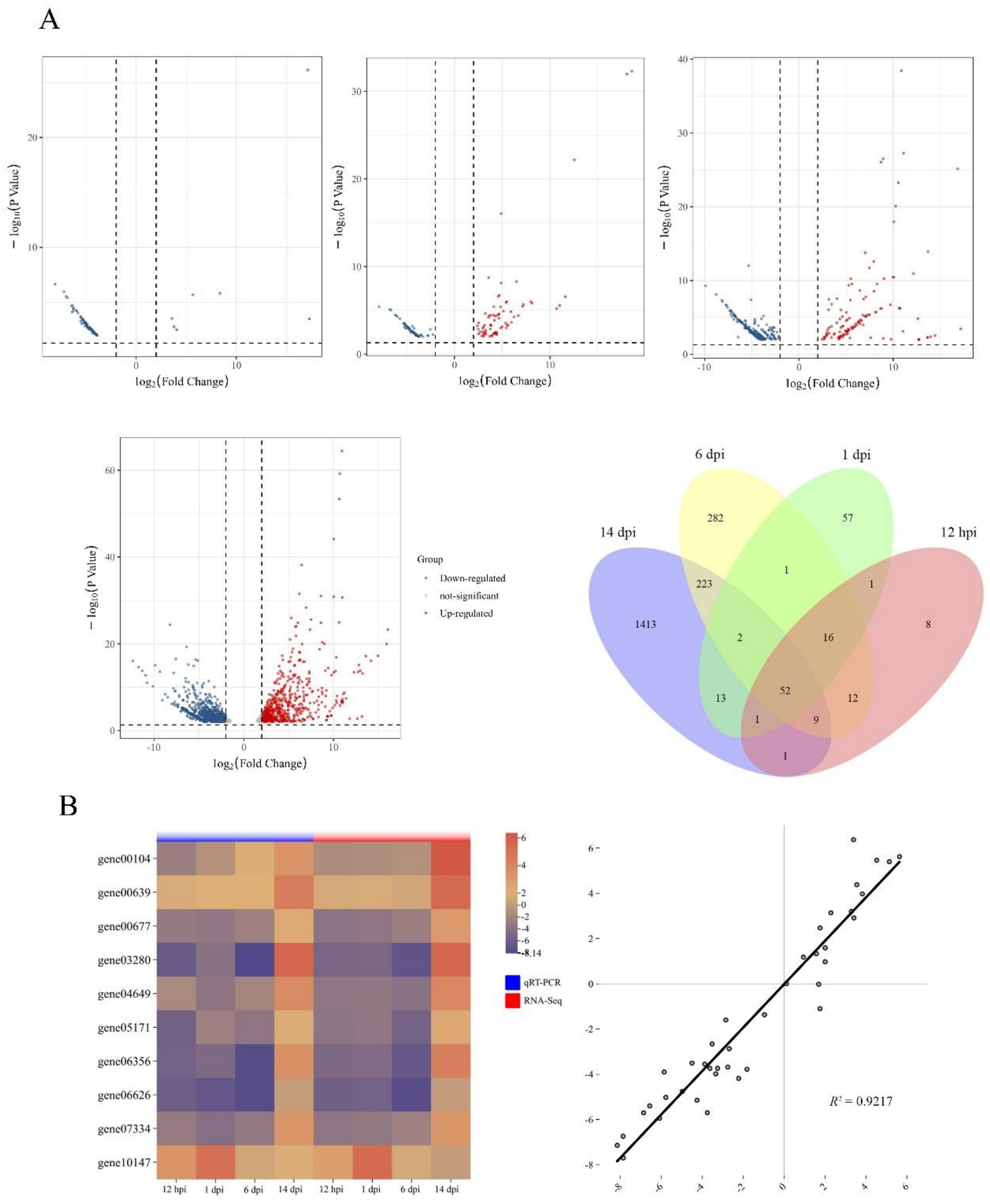

Supplementary Figure 4. (A) Identification of DEGs of *F. nematophilum* strain NQ8GII4 during interaction with alfalfa at 12 hpi, 1 dpi, 6 dpi, and 14 dpi. (B) Validation of RNA-seq data by qRT-PCR. Ten *F. nematophilum* strain NQ8GII4 DEGs were selected for qRT-PCR confirmations in the

same RNA samples used for RNA-seq. The x-axis shows genes validated in this study; The y-axis shows the log2 ratio of F. nematophilum strain NQ8GII4 gene expression in symbiosis vs. control.

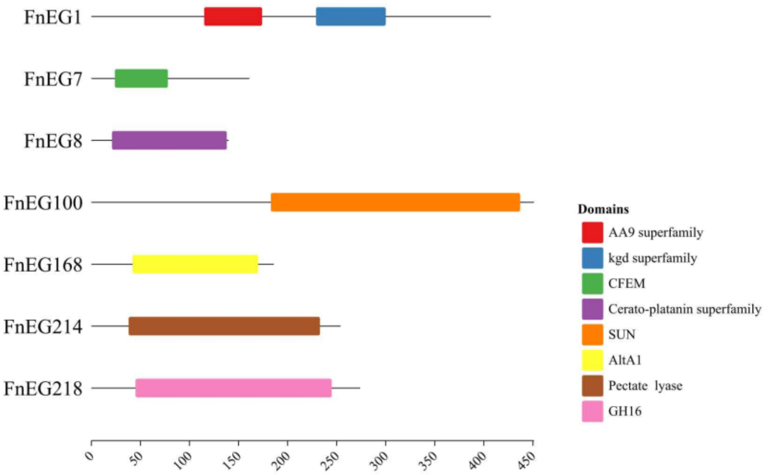

Supplementary Figure 5. Schematic diagram of the protein domains of FnEG1, FnEG7, FnEG8, FnEG100, FnEG168, FnEG214, and FnEG218.

Supplement Table 1. Genomes used in the comparative genomic analyses

Organisms	Taxonomy ID	Strain	Genbank assembly version
Fusarium nematophilum	1053271	NQ8GII4	GCA_033030565.1
Epichloë festucae	877507	Fl1	GCA_003814445.1
Serendipita indica	65672	DSM 11827	GCA_910890315.1
Pestalotiopsis fici	1229662	W106-1	GCA_000516985.1
Fusarium oxysporum	660027	Fo47	GCA_013085055.1
Fusarium oxysporum f. sp. *lycopersici*	426428	4287	GCA_000149955.2
Verticillium dahliae	498257	VdLs.17	GCA_000150675.2
Pyricularia oryzae	242507	70-15	GCA_000002495.2

Fusarium solani	169388	SB1	GCA_023522795.1
Colletotrichum higginsianum	759273	IMI 349063	GCA_001672515.1
Aspergillus nidulans	227321	FGSC A4	GCA_000011425.1
Neurospora crassa	367110	OR74A	GCA_000182925.2

Supplement Table 3. General features of the *F. nematophilum* strain NQ8GII4 genome

Features	*Fusarium nematophilum* NQ8GII4
Genome size (bp)	50,827,403
Coverage (fold)	103×
BUSCOs (%)	97.6
CEGMA (%)	93.95
Number of scaffolds	2,064
N50 (Kb)	144.83
N90 (Kb)	9.09
GC content (%)	53.93
Protein-coding genes	15,149
Gene total length (bp)	28,820,034
Average gene length (bp)	1902.44

Gene density (number of genes per Mb)	307
Average intron number	2.3
Average intron length (bp)	168.57
Average exon number	3.3
Average exon length (bp)	459
tRNA genes	233
Repeat content (%)	5.32

Supplement Table 4. General features of repeat element types in the *F. nematophilum* NQ8GII4 genome

Type	Total length (bp)	No. elements	Percentage in genome (%)
Long terminal repeat (LTR)	138,531	1,094	0.2726
DNA transposons	720,961	2,357	1.4184
Non-LTR retrotransposon (LINEs)	199,653	810	0.3928
Short interspersed repeated (SINE)	2,116	32	0.0042
Roling circle (RC)	367,651	875	0.7233
Unknow	971,848	6,031	1.9121
Total interspersed repeated	2,400,760	11,199	4.7234

Microsatellite DNA	321,471	7,485	0.6325
Satellite DNA	5,194	41	0.0102
Total tandem repeats	326,665	7,526	0.6427

Supplement Table 5. Domain annotation of high-expressed effector in *F. nematophilum* strain NQ8GII4

Gene id	description	Accession
gene05699	AA9	pfam03443
gene06731	AA9	pfam03443
gene00104	AA9 superfamily	cl04076
gene12495	AidA superfamily	cl34620
gene10147	AltA1	pfam16541
gene14033	Amb_all	smart00656
gene05116	BAH/PHD SF superfamily	cd04370/cl22851
gene08050	CBM 4 9 superfamily	cl19911
gene00639	Cerato-platanin superfamily	cl06331
gene09021	COG4913 superfamily/DUF3827 superfamily/PRK14959 superfamily	cl25907/cl28786/cl33044
gene14813	DPBB RlpA EXP N-like	cd22191

gene01131	Egh16-like	pfam11327
gene14474	GH43 AnAbnA-like	cd18831
gene09380	Glyco hydro 12 superfamily	cl03302
gene08152	Glyco hydro 28	pfam00295
gene13004	Glyco hydro 28 superfamily	cl37622
gene06565	Herpes BLLF1 superfamily	cl37540
gene11792	HP	cd07040
gene10314	HRXXH	pfam13933
gene14291	LPMO_AA9	cd21175
gene08860	LpqC	COG3509
gene07433	lyz_endolysin_autolysin/sporang_Gsm superfamily	cd00737/cl41615
gene12208	lyz_endolysin_autolysin/sporang_Gsm superfamily	cd00737/cl41615
gene06694	M35_like superfamily	cl03449
gene14646	NADB_Rossmann superfamily	cl21454
gene13055	NosD superfamily	cl34609
gene01763	Pectate_lyase	pfam03211
gene04716	Pectate_lyase	pfam03211/smart00236

gene07650	Pectate_lyase	pfam03211
gene08057	Pectate_lyase	pfam03211
gene11857	Pectate_lyase	pfam03211
gene13056	Pectate_lyase	pfam03211
gene01431	PelB	COG3866/cl34574
gene13005	PemB superfamily	cl30602
gene13006	PLN02218 superfamily	cl31843
gene05054	PRK10337 superfamily	cl32501
gene14751	SH3 superfamily	cl17036
gene02465	WSC	pfam01822
gene07185	XynA	COG3693
gene06342	YoaJ superfamily	cl27618
